# Topological nodal *i*-wave superconductivity in PtBi_2_

**DOI:** 10.1038/s41586-025-09712-6

**Published:** 2025-11-19

**Authors:** Susmita Changdar, Oleksandr Suvorov, Andrii Kuibarov, Setti Thirupathaiah, Grigory Shipunov, Saicharan Aswartham, Sabine Wurmehl, Iryna Kovalchuk, Klaus Koepernik, Carsten Timm, Bernd Büchner, Ion Cosma Fulga, Sergey Borisenko, Jeroen van den Brink

**Affiliations:** 1 Leibniz Institute for Solid State and Materials Research, IFW Dresden, Dresden, Germany; 2 Institute for Solid State and Materials Physics, TU Dresden, Dresden, Germany; 3 Department of Condensed Matter and Materials Physics, S. N. Bose National Centre for Basic Sciences, Kolkata, India; 4 Kyiv Academic University, Kyiv, Ukraine; 5 Institute of Theoretical Physics, TU Dresden, Dresden, Germany; 6 Würzburg-Dresden Cluster of Excellence ct.qmat, TU Dresden, Dresden, Germany

**Keywords:** Superconducting properties and materials, Topological matter

## Abstract

Most superconducting materials are well understood and conventional—that is, the pairs of electrons that cause the superconductivity by their condensation have the highest possible symmetry. Famous exceptions are the enigmatic high-temperature (high-*T*_c_) cuprate superconductors^1^. Nodes in their superconducting gap are the fingerprint of their unconventional character and imply superconducting pairing of *d*-wave symmetry. Here, by using angle-resolved photoemission spectroscopy, we observe that the Weyl semimetal PtBi_2_ harbours nodes in its superconducting gap, implying unconventional *i*-wave pairing symmetry. At temperatures below 10 K, the superconductivity in PtBi_2_ gaps out its topological surface states, the Fermi arcs, whereas its bulk states remain normal^2^. The nodes in the superconducting gap that we observe are located exactly at the centre of the Fermi arcs and imply the presence of topologically protected Majorana cones around this locus in momentum space. From this, we infer theoretically that robust zero-energy Majorana flat bands emerge at surface step edges. This establishes PtBi_2_ surfaces not only as unconventional, topological *i*-wave superconductors but also as a promising material platform in the ongoing effort to generate and manipulate Majorana bound states.

## Abstract (web summary)

Angle-resolved photoemission spectroscopy shows that Weyl semimetal PtBi_2_ harbours nodes in its superconducting gap, implying unconventional *i*-wave pairing symmetry.

## Main

Electrons in conventional, textbook superconductors, such as lead or niobium, form Cooper pairs with zero angular momentum (*l* = 0) and their pairing symmetry is referred to as *s*-wave. Pairing with higher angular momentum and unconventional superconductivity has been established in cuprate high-temperature superconductors such as YBa_2_Cu_3_O_7_ and Bi_2_Sr_2_CaCu_2_O_8+*x*_. Their *d*-wave pairing (*l* = 2) implies the existence of nodes in the superconducting (SC) gap, locations in momentum space on the Fermi surface where the SC gap vanishes. To establish the presence of these nodes in *d*-wave cuprates, angle-resolved photoemission spectroscopy (ARPES) has played a pivotal part as it can directly map out the size of the SC gap in momentum space^3–7^.

Although there is substantial theoretical work discussing SC states with pairing symmetry beyond *l* = 2, at present, there is no spectroscopic evidence for unconventional superconductivity beyond *d*-wave^8–11^. This makes our ARPES-based observation of nodal superconductivity on the Fermi arcs of PtBi_2_ stand out because a symmetry analysis of its nodal structure implies that the gap here exhibits *i*-wave symmetry (*l* = 6). As Fermi-arc states are chiral and nondegenerate, this sign change in the SC order parameter along the arc implies the formation of a surface Majorana cone, similar to the Majorana cones expected to occur on the surface of three-dimensional (3D) strong topological superconductors^12^ or ^3^He (ref. ^13^), rendering PtBi_2_ a topological superconductor. This is remarkable because materials with intrinsic topological superconductivity are scarce. So far, candidate materials include Sr_2_RuO_4_ (ref. ^14^), transition-metal dichalcogenides such as T_d_-MoTe_2_ (ref. ^15^) and 4H_b_-TaS_2_ (ref. ^16^), uranium-based heavy-fermion systems^17,18^, β-PdBi_2_ (refs. ^19–22^), and very recently, the kagome material RbV_3_Sb_5_ (ref. ^23^). In these systems, however, different experimental methods produce inconclusive and sometimes contradictory results, so that to date no material has been convincingly shown to be an intrinsic topological superconductor^24^.

The unconventional *i*-wave SC order implies the presence of six Majorana cones on a given PtBi_2_ surface, each with its own topological invariant—a winding number equal to either +1 or −1. Symmetry dictates that all six have the same winding number. This is the signature of a quantum anomaly: on the opposite surface of a SC slab, there are six Majorana modes of opposite winding number, ensuring that the sum over all topological invariants vanishes. We will show theoretically that the edge-state structure related to these topological nodes causes the existence of zero-energy, dispersionless Majorana modes localized at the sample hinges, which in practice may be realized by sufficiently high step edges at the surface.

## ARPES characterization

Trigonal PtBi_2_ is a noncentrosymmetric Weyl semimetal belonging to space group *P*31*m* (ref. ^25^). Its electronic structure hosts 12 Weyl cones, related to each other by time reversal as well as threefold rotation symmetry, which are positioned about 47 meV above the Fermi level^2,26–28^. On cleaving, two different types of surfaces are produced, a kagome-type surface and a decorated honeycomb surface^2,27^. At both terminations, scanning tunnelling spectroscopy has established the presence of superconductivity^28^, carried by the topological surface states of this Weyl semimetal—the Fermi arcs—which gap out at temperatures below 10 K (ref. ^2^).

**Fig. 1:**
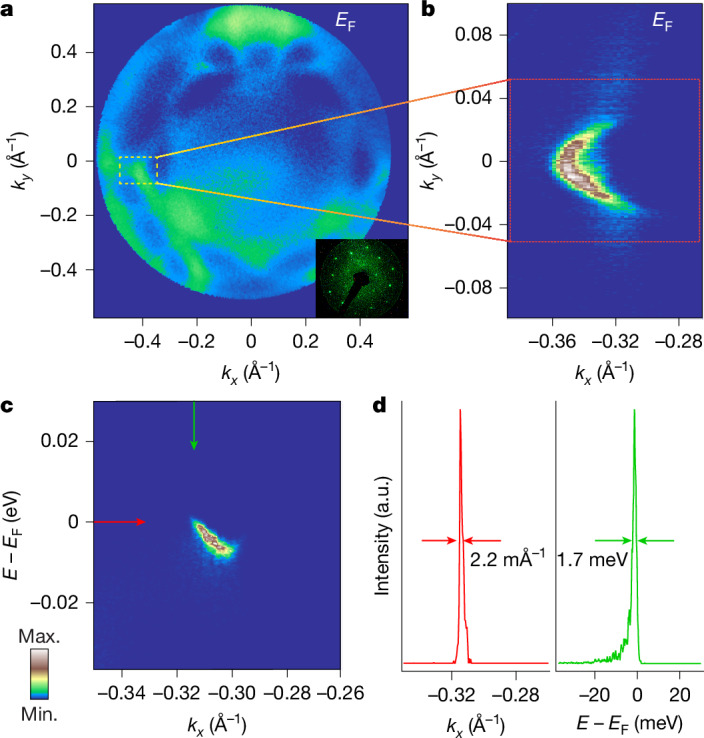
Progress in experimental accuracy. **a**, Fermi surface map observed with a FeSuMa and He-I lamp with *hν* = 21.2 eV from kagome-type termination. Inset, collected low-energy electron diffraction image on PtBi_2_ single crystal. The yellow box marks the position of the arc on the Fermi surface map. **b**, The arc becomes well resolved in the Fermi surface observed with laser ARPES with *hν* = 6 eV (kagome-type termination). **c**, Momentum–energy intensity distribution corresponding to the momentum cut through the arc (decorated honeycomb termination). **d**, Momentum distribution curve and energy distribution curve plotted along the red and green arrows in **c**.

We have carried out ARPES experiments with improved resolution to specifically study the gap function on the Fermi arcs of PtBi_2_. We start by demonstrating in Fig. 1 the progress in experimental accuracy in comparison with our previous study^2^. It has already been shown that the most precise measurements can be carried out with the lowest possible photon energy, which leads to the lowest kinetic energy of the photoelectrons of interest. However, these low kinetic energies correspond to relatively small values of the absolute momentum, which do not cover a sufficient portion of the Brillouin zone. Therefore, we first perform the Fermi surface mapping using the higher photon energies available in the laboratory (21.2 eV from a helium lamp) and use for this purpose a FeSuMa (Fermi surface mapper) analyser^29^, which allows us to record these maps with isotropic angular resolution. This dataset, together with the low-energy electron diffraction picture, is shown in Fig. 1a.

Although the presence of arcs along Γ–M is visible on the map, the arcs themselves are not well pronounced at this photon energy, in agreement with our previous observation at a synchrotron^2^. After checking the quality of the surface and orientation of the sample, we use a laser source with *hν* = 6 eV to enlarge in to the vicinity of the M point of the Brillouin zone and collect ARPES data with better momentum and energy resolution using a conventional analyser (Fig. 1b). With this method, the arc is seen with unprecedented clarity and in close agreement with the DFT slab calculations, for example, in ref. ^2^. Another advantage of using this particular photon energy is the strong enhancement of the arc intensity compared with the bulk bands. The same holds when considering a momentum–energy cut through the arc, as shown in Fig. 1c. There are basically no other features visible except for the surface band supporting the Fermi arc. Instead of the parabolic dispersion usually underlying closed electron-like pockets of the Fermi contours, we directly observe an asymmetric shape, just as expected from the open nature of the Fermi arcs in Weyl semimetals.

We further reduce the dimension of the dataset by extracting the momentum distribution curve and the energy distribution curve along the red and green arrows in Fig. 1c, respectively. The resulting very sharp and strong peaks are shown in Fig. 1d. Both full-width at half maximum (FWHM) values, namely, 2.2 mÅ^−1^ and 1.7 meV, are the smallest in the history of photoemission from solids for these lineshapes, to our knowledge. Thus, the Fermi arcs in PtBi_2_ appear to represent extraordinary electronic states, strongly localized in terms of energy, momentum and space. The ability to detect these features with the precision shown above provides an opportunity to investigate the order parameter when the arcs become superconducting.

## Evidence for nodes in the SC gap

**Fig. 2:**
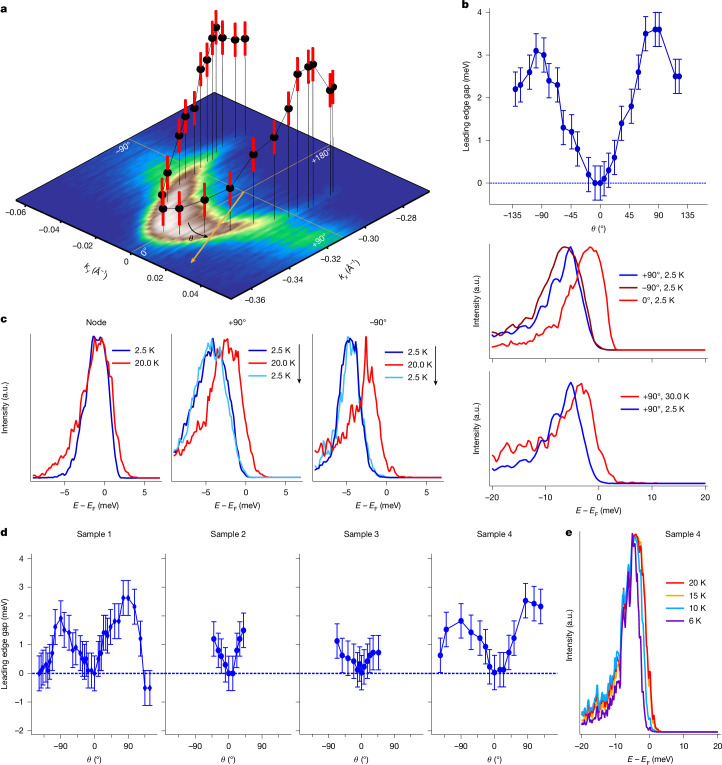
Anisotropic superconducting gap. **a**, Leading edge gap across different points of the arc (kagome-type termination). **b**, Angular dependence of the gap, showing a node at *θ* = 0° and a maximum gap at ±90° for cleave 1 of sample 1 (top). The middle panel shows the leading edge gap from energy distribution curves taken along *θ* = 0° and ±90° at 2.5 K. This is equivalent to the gap observed from energy distribution curves taken at +90° at 2.5 K and 30 K (bottom). **c**, Energy distribution curves taken at the node, +90° and −90°, respectively, at 2.5 K and 20 K. For ±90°, the temperature is cycled back to 2.5 K, which overlaps with the initial 2.5 K energy distribution curves. **d**, Angular dependence for cleave 2 of sample 1 (left) and for three other PtBi_2_ single crystals from different batches. **e**, Temperature dependence of the energy distribution curve corresponding to the arc exhibiting the gradual closing of the leading edge gap at higher temperature. Error bars in **a**, **b** and **d** show standard deviation and represent uncertainties in determining Fermi momenta and statistical errors of the leading edge gap from the fitting procedure.

Next, we focus on determining the leading edge gap at different points along the arc in Fig. 2. The first observation is that this gap is not isotropic. The three-dimensional (3D) image in Fig. 2a shows the points of the arc at which the leading edge gap is determined and how the gap changes along the arc. The momentum *k*_*y*_ = 0 Å^−1^ corresponds to the Γ–M direction in the Brillouin zone. The gap is also plotted in Fig. 2b, showing this anisotropy. An immediate and rather surprising observation is that the leading edge gap seems to close when the arc crosses the Γ–M line, indicating the existence of a node. Owing to the finite resolution, it is not possible to determine the exact behaviour of the gap function very close to this point, as is the case for the high-*T*_c_ cuprates, but our temperature-dependent measurements also confirm nodal behaviour. In Fig. 2c, we show energy distribution curves taken from the node (0°) and ±90° along the arc above and below the critical temperature. As suggested by the gap function Fig. 2b (top), the gap increases with distance from the node, resulting in a shift of the coherence peak.

The gap reaches its maximum at approximately *θ* = ±90° and then starts to decrease again at higher *θ* (Fig. 2b). In Fig. 2b (middle), we plot the energy distribution curves taken along the yellow lines marked as *θ* = 0° and ±90° in Fig. 2a, which correspond to these maxima. The coherence peak shifts by about 3.6 meV towards higher kinetic energy for *θ* = ±90° compared with *θ* = 0°. This value is in close agreement with our earlier study and other experiments (see ref. ^2^ and references therein).

We also note the apparent presence of plateaus at *θ* = ±45°, which could be an indication of the admixture of even higher orders (see, for example, ref. ^30^); however, at the current accuracy, we cannot rule out that this feature is a robust observation beyond the error bars.

The gap is also observed from the energy distribution curves taken above and below the critical temperature *T*_c_ along *θ* = +90° (Fig. 2b, bottom). To further confirm the existence of the node on the arc, we have repeated the measurements for four different PtBi_2_ samples grown in different batches. All data presented in Fig. 2a,b were taken from sample 1, cleave 1. The data presented in Fig. 2c were taken from sample 4. The angular dependence of the gap for cleave 2 of sample 1 is presented in the leftmost panel of Fig. 2d. As expected, the angular dependence of the gap is quite similar to cleave 1 (Fig. 2b) with its maximum of about 2.5 meV at *θ* = ±90°. Apart from the node at 0°, the leading edge gap gradually decreases and closes again at ±125°. As the arc blends with the bulk bands at higher *θ*, the effect of superconductivity, which is intrinsic to the arc, starts to disappear. For further validation of our observation of the node at 0°, we have repeated the same measurement on three other PtBi_2_ samples. Samples 2, 3 and 4 exhibit a node at the same position as sample 1, as seen in Fig. 2d. Moreover, we performed temperature-dependence measurements on sample 4, which shows the gradual closure of the gap with temperature (Fig. 2e). The gap decreases as we increase temperature from 6 K to 10 K but remains open. At 15 K, the gap seems to be closed as we do not observe any peak shift between 15 K and 20 K. Hence, the critical temperature is within the range of 10–15 K, as established in earlier studies^2^.

It is important to note that the extracted leading edge position from any energy distribution curve is influenced by energy–momentum resolution, position of the Fermi level and Fermi function (that is, temperature of the sample). These effects can be minimized by tracking the peak position or the trailing edge position of the same energy distribution curves (see section ‘Leading edge, peak, and trailing edge positions’ and Extended Data Fig. 3b) along the arc as they are located away from the Fermi level, at higher binding energy. This point is further elaborated in the Methods section ‘Leading edge, peak, and trailing edge positions’.

## SC pairing symmetry

Based on the *C*_3*v*_ point group of trigonal PtBi_2_, the possible SC states can be classified according to their irreducible representations (irreps) *A*_1_, *A*_2_ and *E*. States belonging to the trivial irrep *A*_1_ are invariant (even) under all point-group operations, so symmetry does not impose gap nodes. SC states of *E* symmetry must break either rotation symmetry (and then can have nodes on some but not all Fermi arcs) or time-reversal symmetry (and are nodeless), see Methods. There is no experimental evidence for the SC state breaking time-reversal symmetry, nor for the simultaneous presence of fully gapped and nodal Fermi arcs, speaking against *E* symmetry. This leaves SC states of *A*_2_ symmetry, which have symmetry-imposed gap nodes at the arc centres and the same gap profile for all arcs. For the *A*_2_ irrep, the lowest-order time-reversal-symmetric basis function of the polar angle *ϕ* in two-dimensional (2D) momentum space is sin *Iϕ* with *l* = 6, implying *i*-wave pairing symmetry. As the Fermi arc is chiral and nondegenerate, a sign change of the SC order parameter along the arc directly produces a surface Majorana cone, the hallmark of topological superconductivity. This is similar to the Majorana cones that are expected to occur on the surface of 3D strong topological SCs (ref. ^12^) or ^3^He (ref. ^13^).

## Comparison with electronic structure calculations

To compare the ARPES results with density functional theory (DFT), we modified the approach of ref. ^2^ to include nodal gap functions. In particular, we use in our DFT Wannier model *i*-wave pairing of the form *V*_0_ sin(6*ϕ*), expanded for small momenta around the node (Methods). We restrict pairing and thus *V*_0_ ≠ 0 to the surface block of a semi-infinite slab and solve the Bogoliubov–de-Gennes (BdG) equations in the semi-infinite slab geometry to obtain the surface Bloch spectral density ${A}_{{\rm{bl}}}({\bf{k}},E)$.

**Fig. 3:**
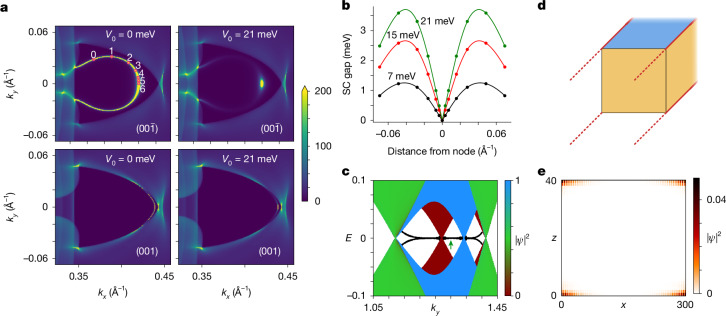
Calculated properties of the *i*-wave superconductor—Majorana cones and hinge states. **a**, Calculated spectral density at the Fermi level in a DFT-BdG Wannier model for the $(00\bar{1})$ surface (decorated honeycomb termination^27^, top panels) and for the (001) surface (bottom panels) with superconducting *i*-wave pairing *V*_0_ sin(6*ϕ*) on the first three surface layers for coupling strengths *V*_0_ = 0 meV (no superconductivity) and *V*_0_ = 21 meV. The points at which the gap was determined are indicated in the normal-state panel using labels 0–6. Spectral weights larger than 200 are shown in yellow. **b**, The SC gap as a function of distance from the node for three coupling strengths *V*_0_ = 7 meV (black), 15 meV (red) and 21 meV (green), in the case of the decorated honeycomb termination, as in the top panels of **a**. Circles are calculated values, the lines are guides to the eye. The SC gap for the other termination is shown in the Methods. **c**, Electronic structure of the effective model. The colour scale denotes the probability density of the states in real space. Bulk Weyl cones are shown in green, top Majorana cones are shown in red and bottom Majorana cones are shown in blue. Dispersionless zero-energy Majorana hinge modes are shown in black. See the Methods for details of the simulation. **d**, Sketch of the prism geometry used in the effective model. The system is infinite in the *y-*direction and finite in the *x*- and *z*-directions. The superconducting top surface is shown in blue, and the Majorana hinge modes are shown in red. **e**, Probability density of the four Majorana hinge modes in the prism geometry of **d**, computed for *k*_*y*_ = 1.3, corresponding to the green arrow in **c**.

Figure 3a shows the results for different coupling strengths *V*_0_. In the normal state (*V*_0_ = 0), the gap vanishes along the whole Fermi arc, whereas for finite *V*_0_, the gap closes at *k*_*y*_ = 0, the centre of the Majorana cone. Owing to a numerically finite lifetime of 0.05 meV, a remnant spectral weight is visible along the arc, especially for small *V*_0_. The gap, however, is finite, except at the node.

The gap was determined by magnifying the points **k**_*i*_, indicated in the normal state panel, to locate points exactly on top of the arc, followed by a scan of *A*_bl_(**k**_*i*_, *E*) to determine the quasiparticle edges. Comparing the resulting gaps, shown in Fig. 3b, with the experiment allows us to deduce a coupling strength of *V*_0_ = 15–20 meV. The gap on the arc has two maxima that are caused by the gap vanishing at both the Γ–M node and the surface-projected Weyl node position, at which the arc states merge with the bulk. Even when we include in the calculations only the lowest *i*-wave harmonic, the position of the gap maxima at 0.04 Å^−1^ is close to the experimental value 0.06 ± 0.01 Å^−1^.

## Step-edge Majorana modes

To further explore the implications of the presence of the Majorana cones theoretically, we use a simplified effective tight-binding model for PtBi_2_ that captures both the lattice symmetries and the relative positions of the Weyl cones in the normal state^27^ (see also the Methods). The model disregards topologically trivial bands crossing the Fermi level while ensuring a marked separation between Weyl cones in momentum space.

Without surface superconductivity, the projections of the 12 Weyl cones are connected pairwise by surface Fermi arcs. On including the surface pairing terms with *A*_2_ symmetry in a slab geometry (infinite along the *x-* and *y-*directions, finite along the *z*-direction), the Fermi arcs on both the top and the bottom surfaces are gapped out by the pairing, leading to the formation of gapless Majorana cones along the Γ–M direction of the slab Brillouin zone (Extended Data Fig. 2b). As in the DFT-BdG description, each Fermi arc produces a surface Majorana cone, such that there exist six cones on the top surface and six on the bottom surface. In the presence of both time-reversal and particle–hole symmetries, corresponding to class DIII in the Altland–Zirnbauer classification^31^, each individual Majorana cone is topologically protected, being characterized by a nonzero winding number, taking the value ±1 (ref. ^32^) (Methods). Note that this scenario is distinct from that of gapped strong topological superconductors in class DIII, in which the surface hosts an integer number of Majorana cones because of 3D bulk topology. Here, instead, the bulk remains metallic and does not superconduct, whereas the surface realizes a 2D gapless topological phase^33^. As the surface Majorana cones on a given surface are related to each other by threefold rotation and/or time-reversal symmetry, they all have the same sign of the winding number^33^, which in our case is −1 for the top surface and +1 for the bottom surface. Thus, each surface of the system forms a so-called anomalous topological superconductor (Methods), one in which the sum of Majorana-cone winding numbers does not cancel, and therefore one which is impossible to realize in a purely 2D system. By contrast, in the gapless topological phases of purely 2D superconductors, the winding numbers of Majorana cones must vanish, and the number of cones must be a multiple of four, as shown in ref. ^33^.

Breaking time-reversal symmetry removes the topological protection of the Majorana cones, which then acquire a gap (for details, see the Methods). This implies that this weak magnetic field enhances the surface SC gap close to the node, while reducing it for other momenta along the arc, a prediction that may be tested experimentally. When time-reversal symmetry is preserved, however, the nonzero winding number of the surface Majorana cones necessarily implies the existence of zero-energy Majorana modes^34^ localized at the boundaries of the surface, that is, at the hinges of the 3D system. This symmetry-based observation is confirmed in Fig. 3c-e by a calculation on an infinite prism geometry [infinite along the *y*-direction and finite along the *x-* and *z-*directions, as shown in Fig. 3d). The prism band structure (Fig. 3c) contains the projection of two Majorana cones located on the top surface, which overlap in *k*_*y*_ in the prism Brillouin zone (shown in blue, total winding number + 2), and two Majorana cones located on the bottom surface (red, winding number −2). At *k*_*y*_ values between them (Fig. 3c, green arrow), there appear four degenerate zero modes, shown in black, which are localized at the hinges of the infinite prism (Fig. 3e). Outside this momentum range, the hinge modes are no longer topologically protected, such that they can hybridize and split away from zero energy. Similar to the surface Majorana cones themselves, the hinge modes will move away from the Fermi level under a Zeeman field, providing another signature of topological SC that is experimentally accessible in local measurements at hinges or large step edges of PtBi_2_.

Our observation of nodal superconductivity in the Fermi arcs of PtBi_2_ raises the question as to what drives this unconventional *i*-wave pairing. Although in cuprates the mechanism for high-temperature *d*-wave superconductivity remains under debate, there is consensus that the presence of strong electron–electron interactions stabilizes the nodal *d*-wave pairing channel over the nodeless *s*-wave channel. In PtBi_2_, the electronic states are highly delocalized in nature and strong electronic correlations are not expected, and are as yet without experimental indication. By contrast, the topological character of the superconducting Fermi arcs sets PtBi_2_ apart from any other known superconductor to date. The mechanism by which an *i*-wave superconductor emerges from pairing of these topological states is yet to be established.

Finally, we note that the coexistence of gapless Majorana cones with a metallic bulk impedes the applicability of PtBi_2_ towards quantum computation, at least in its current form. This can potentially be mitigated by the fabrication of ultrathin samples: when the thickness of the material becomes small enough, the contribution of unwanted gapless bulk modes will be reduced, or even eliminated. Another potential manipulation may involve breaking time-reversal symmetry to gap out the surface Majorana cones in such a way as to leave behind either chiral Majorana edge modes or zero-dimensional Majorana bound states localized at the corners of the material. Both types of gapless mode have been proposed as a potential avenue towards topological quantum computation^35,36^. Alternatively, we might also predict controlling the phase difference between the top- and bottom-surface superconductors, realizing a planar Josephson junction that provides an avenue towards quantum computation^37^.

## Methods

### DFT calculations and BdG model

We performed DFT calculations using the full-potential local orbital code FPLO^38^ within the generalized gradient approximation^39^, using the tetrahedron method with 12^3^ points for the Brillouin integration. Subsequently, a maximally projected symmetry-conserving Wannier model^40^ containing Wannier orbitals for each Bi 6*p* and Pt 6*s*, 5*d* basis orbital was constructed. The model is mapped onto a semi-infinite slab with a surface block consisting of three Bi_6_Pt_3_ layers on which only a nonzero gap function is added. In detail, the gap function reads

$$\Delta ={\delta }_{\text{orbital-qns}}{\rm{i}}{\sigma }_{y}D(k){V}_{0}$$

with $D(k)=1,020{k}_{x}{k}_{y}(3{k}_{x}^{4}-10{k}_{x}^{2}{k}_{y}^{2}+3{k}_{y}^{4})$ being a scaled Taylor expansion of sin(6*ϕ*). The scaling factor was chosen to fulfil *D*(*k*) = 1 at *k* = (0.4, 0.0325) Å^−1^. The Bloch spectral density for a penetration depth of three surface blocks is obtained by solving the BdG equations using Green function recursion^2,41^ in this semi-infinite geometry.

**Extended Data Fig. 1:**
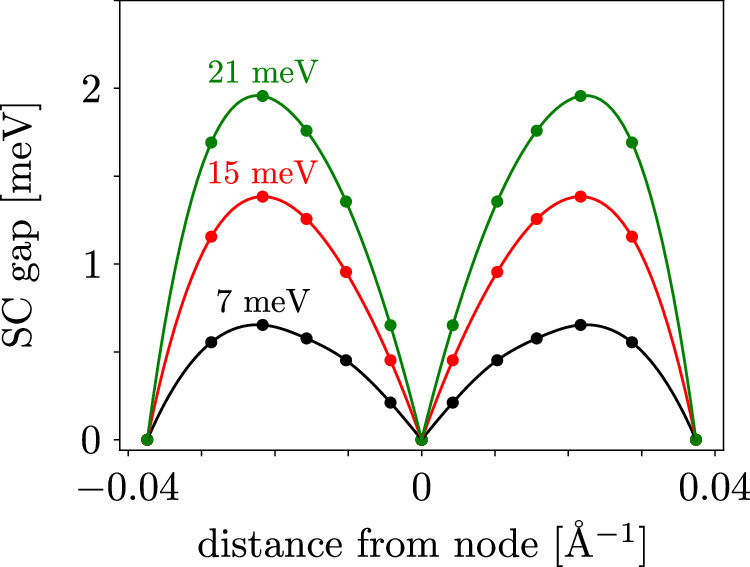
Superconducting gap variation. The SC gap as a function of distance from the node for *V*_0_ values of 7 (black), 15 (red), and 21 meV (green). This figure is for the (001) termination and shows the same features as Fig. 3(b) of the main text.

In Extended Data Fig. 1, we show results for the SC gap on the (001) surface as a function of the distance from the node for different values of *V*_0_ (compare with Fig. 3b). Also, for this termination, we observe the same features: a V-shaped gap that increases with *V*_0_.

### Symmetry-allowed SC states

As noted in the main text, the symmetry of possible SC order parameters can be classified in terms of the irreps *A*_1_, *A*_2_ and *E* of the point group *C*_3*v*_ of PtBi_2_. For trigonal PtBi_2_, the mirror planes contain the Γ–K lines and not the Γ–M lines, and thus they do not include the centre points of the Fermi arcs, at which the apparent nodes are located. However, time-reversal symmetry acts like twofold rotation symmetry about the *z*-axis for momenta **k** = (*k*_*x*_, *k*_*y*_). Consequently, any time-reversal-symmetric function of **k** that is even under all mirror reflections of the lattice is also even, and that which is odd is also odd, under mirror reflections with respect to vertical planes through the arc centres.

Owing to the lack of inversion symmetry, spin-singlet and spin-triplet pairing generically mix, and it is necessary to consider 2 × 2 pairing matrices *Δ*(**k**) appearing in the BdG Hamiltonian

1 $${\mathcal{H}}({\bf{k}})=\left(\begin{array}{rc}{H}_{N}({\bf{k}}) & \varDelta ({\bf{k}})\\ {\varDelta }^{\dagger }({\bf{k}}) & -{H}_{N}^{{\rm{T}}}(-{\bf{k}})\end{array}\right).$$

To construct possible pairing matrices *Δ*(**k**), we have to consider the symmetry properties of **k**-dependent form factors and of matrices acting on spin space.

The **k**-dependent factors are relevant only on the Fermi lines. To parameterize them, we start from the polar angle *ϕ* of **k**. Electronic bands are continuous, so that the Fermi arcs are connected by the Fermi surfaces of bulk states. As the Fermi arcs are horseshoe-shaped, the Fermi surface is not convex, and the points on the arcs are not uniquely labelled by *ϕ*. This can be resolved by deforming the parameterization without changing its symmetry and is irrelevant for our analysis. The lowest-order basis functions of *ϕ* together with their irrep and their sign under time reversal are listed in Extended Data Table 1. Basis functions of higher order modulate only solutions that can already be constructed from them.

A basis of the space of 2 × 2 matrices is given by the identity matrix *σ*_0_ and the Pauli matrices *σ*_*x*_, *σ*_*y*_ and *σ*_*z*_. These transform as irreducible tensor operators of the irreps, as shown in Extended Data Table 2.

By multiplying the form factors and the basis matrices, we can obtain all possible SC states. Using standard rules for products of irreps, we can choose them to belong to specific irreps. However, only products that are even under time reversal satisfy fermionic antisymmetry *Δ*^T^(−**k**) = −*Δ*(**k**) (ref. ^42^).

The SC state of full symmetry, that is, belonging to *A*_1_, was considered in ref. ^27^. The irrep *A*_1_ is even under all mirror reflections and rotations, and thus *A*_1_ symmetry does not impose any gap nodes.

The irrep *E* is 2D, leading to a two-component SC order parameter. The first component, by itself as well as any symmetry-related SC states, is odd under some mirror reflections. This imposes nodes at some arc centres but not at all of them, thereby breaking the threefold rotation symmetry. The second component, by itself and symmetry-related states, does not impose any nodes. This follows from the observation that any order parameter belonging to the second component of *E* can be constructed from an *A*_1_ (full symmetry) order parameter by multiplication by cos 2*ϕ*, which does not have zeros on the arcs. The gap magnitude is not the same at all arcs, so that such a state also breaks rotation symmetry. We do not find experimental indications for this symmetry breaking. There are also time-reversal-symmetry-breaking *E* states, constructed by superposition of the two components with a phase shift of ±π/2. These states do not have nodes. Moreover, there is no experimental evidence for broken time-reversal symmetry.

The remaining irrep *A*_2_ is odd under all mirror reflections. This fact imposes nodes at all arc centres. In particular, it is odd under *ϕ* ↦ −*ϕ*. Owing to the preserved rotation and time-reversal symmetries, the gap profile is the same for all arcs. Hence, only *A*_2_ pairing symmetry is consistent with nodes at the arc centres.

Constructing the possible pairing matrices as described above, we obtain

2 $$\begin{array}{l}\varDelta (\phi )\,=\,[{f}_{1}(\cos \phi \,{{\sigma }}_{x}+\sin \phi \,{{\sigma }}_{y})+{f}_{3}\sin 3\phi \,{{\sigma }}_{z}\\ \,\,\,\,+\,{f}_{5}(\cos 5\phi \,{{\sigma }}_{x}-\sin 5\phi \,{{\sigma }}_{y})+{f}_{6}\sin 6\phi \,{{\sigma }}_{0}]\,{U}_{T},\end{array}$$

where the coefficients *f*_1_, *f*_3_, *f*_5_ and *f*_6_ can be chosen real because of time-reversal symmetry. *U*_*T*_ = i*σ*_*y*_ is the unitary part of the time-reversal operator. The four terms describe *p*-wave (*l* = 1) in-plane spin-triplet pairing, *f*-wave (*l* = 3) out-of-plane spin-triplet pairing, *h*-wave (*l* = 5) in-plane spin-triplet pairing and *i*-wave (*l* = 6) spin-singlet pairing, respectively. The SC energy gap on the Fermi arcs is a real function of *ϕ*. As the BdG Hamiltonian with the pairing matrix in equation (2) preserves threefold rotation and time-reversal symmetries, so does the energy gap. Moreover, we have shown above that it must be odd under reflection at the arc centres. Hence, the energy gap is a basis function belonging to the irrep *A*_2_, proportional to sin 6*ϕ* plus higher harmonics of the same symmetry, that is, it has *i*-wave form.

### Anomalous topological superconductivity

Two-dimensional superconducting systems that obey time-reversal symmetry (class DIII in the Altland–Zirnbauer table^31^) may host gapless Majorana cones in their 2D Brillouin zone. These gapless points are in many respects analogous to the Weyl points of 3D crystals, with the important exception that they require chiral symmetry to remain protected. They are characterized by an integer topological invariant (here, a winding number instead of the Chern number associated with Weyl points) and always occur in pairs. Furthermore, time-reversal symmetry relates cones with opposite momenta and the same integer invariant, just as with Weyl cones. Thus, as the total topological invariant associated with all band crossings must vanish in a periodic Brillouin zone, Majorana and Weyl cones must come in multiples of four as long as time-reversal symmetry is preserved.

In PtBi_2_, instead, our results indicate the presence of six Majorana cones on a single superconducting surface, violating the above requirement. Moreover, all six cones have the winding numbers of the same sign, such that their sum does not add up to zero. The resolution of this apparent paradox is that other Majorana cones occur on the opposite surface of the crystal. Taking both surfaces into account, the total number of Majorana cones is 12 (a multiple of four), and the sum of all winding numbers vanishes.

This is the sense in which we state that the 2D superconductor forming on the surface of PtBi_2_ is anomalous. Given their multiplicity and winding number, their Majorana cones cannot occur in a purely 2D, standalone superconducting state, but only as surface modes of a higher-dimensional, 3D system. This is analogous to the unidirectional edge modes of quantum Hall systems, which cannot be realized as standalone 1D systems but only as edge states.

### PtBi_2_ tight-binding model calculations

We explore the consequences of surface Majorana cones using the toy model recently introduced in ref. ^27^, the properties of which we briefly summarize below. It is defined on a trigonal lattice, with Bravais vectors **a**_1_ = (0, 1, 0), ${{\bf{a}}}_{2}=(\sqrt{3}/2,-1/2,0)$, **a**_3_ = (0, 0, 1), and consists of two spinful orbitals per unit cell. Setting *k*_*i*_ = **a**_*i*_ ⋅ **k**, the momentum-space Hamiltonian reads

3 $$\begin{array}{l}H({k}_{1},{k}_{2},{k}_{3})=[\,\mu -t\,\cos {k}_{1}-t\,\cos {k}_{2}-t\,\cos ({k}_{1}+{k}_{2})]{\varGamma }_{1}\\ \,\,+\beta (\cos {k}_{3}\,{\varGamma }_{1}+\sin {k}_{3}\,{\varGamma }_{3})\\ \,\,+\lambda [\sin {k}_{1}+\sin {k}_{2}-\sin ({k}_{1}+{k}_{2})]\,{\varGamma }_{3}\\ \,\,+\alpha (1-\cos {k}_{3})[\sin {k}_{1}\,{\varGamma }_{2}+\sin {k}_{2}\,{\varGamma }_{2,1}-\sin ({k}_{1}+{k}_{2})\,{\varGamma }_{2,2}]\\ \,\,+\gamma {\tau }_{x}{\sigma }_{0},\end{array}$$

with *Γ*_1_ = *τ*_*z*_*σ*_0_, *Γ*_2_ = *τ*_*x*_*σ*_*x*_, *Γ*_3_ = *τ*_*y*_*σ*_0_, where Pauli matrices *τ*_*x*_, *τ*_*y*_ and *τ*_*z*_ encode the orbital and Pauli matrices *σ*_*x*_, *σ*_*y*_ and *σ*_*z*_ denote spin, and

4 $${\varGamma }_{2,j}={{\mathcal{C}}}_{3}^{j}{\varGamma }_{2}{{\mathcal{C}}}_{3}^{-j},$$

where

5 $${{\mathcal{C}}}_{3}={\tau }_{0}\,\exp \left(-{\rm{i}}\,\frac{{\rm{\pi }}}{3}\,{\sigma }_{z}\right)$$

represents threefold rotations around **a**_3_, that is, around the *z*-axis. Besides the threefold rotation,

6 $${{\mathcal{C}}}_{3}^{-1}H({k}_{1},{k}_{2},{k}_{3}){{\mathcal{C}}}_{3}=H({k}_{2},-{k}_{1}-{k}_{2},{k}_{3}),$$

the model also reproduces the other lattice symmetries of PtBi_2_. There is a mirror symmetry along the *k*_1_ = −2*k*_2_ plane of the Brillouin zone, with *M*_1_ = i*τ*_0_*σ*_*x*_,

7 $${M}_{1}^{-1}H({k}_{1},{k}_{2},{k}_{3}){M}_{1}=H({k}_{1},-{k}_{1}-{k}_{2},{k}_{3})$$

and the two other mirror planes are obtained by applying the threefold rotation symmetry. Furthermore, the model obeys time-reversal symmetry $T={\rm{i}}{\tau }_{0}{\sigma }_{y}{\mathcal{K}}$ with the complex conjugation ${\mathcal{K}}$ such that *TH*(*k*_1_, *k*_2_, *k*_3_)*T*^−1^ = H^*^(−*k*_1_, −*k*_2_, −*k*_3_).

We choose the hopping amplitude *t* = 1 as the energy unit and express all other energy scales relative to it. In all numerical simulations, we have used the parameters *μ* = 2, *β* = −0.75, *λ* = 3, *α* = 0.75 and *γ* = 0.5, for which the band structure hosts 12 well-separated Weyl cones, thus reproducing the behaviour of PtBi_2_.

**Extended Data Fig. 2:**
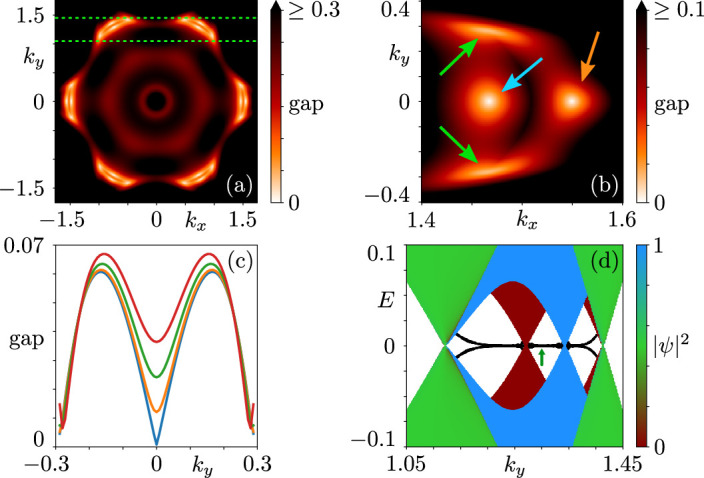
Majorana cones and hinge states from effective model description. **(a)** Energy gap as a function of the dimensionless momenta *k*_*x*_ and *k*_*y*_ in a slab geometry, finite in the *z* direction. The projections of the Weyl cones are connected pairwise by two weakly-gapped Fermi arcs, one on the top surface and one on the bottom surface. **(b)** Closeup showing two Weyl cone projections (green arrows), a Majorana cone on the bottom surface (blue arrow), and a Majorana cone on the top surface (orange arrow). **(c)** Gap along the Fermi arc on the top surface. An out-of-plane Zeeman field gaps out the Majorana cone, enhancing the SC gap. Blue, orange, green, and red curves correspond to increasing Zeeman fields *V*_*z*_ = 0, 0.05, 0.1, and 0.15, respectively. **(d)** Bandstructure of the system in a prism geometry, infinite along *y*, and finite along *x* and *z*, plotted for the values of *k*_*y*_ between the two green dashed lines of panel (a). This panel appears also in the main text. The color scale denotes the probability density of the states in real space. Bulk Weyl cones are shown in green, and top and bottom Majorana cones are shown in red and blue, respectively. Dispersionless zero-energy Majorana hinge modes are shown in black.

The numerical results shown in Extended Data Fig. 2 are obtained for systems that have a finite number of unit cells in the *z-*direction (either in a slab geometry or in a prism geometry), by adding SC pairing to the two top-most and the two bottom-most unit cells. The BdG Hamiltonian takes the same block form as equation (1), with the upper-diagonal block given by equation (3). As all pairing terms with *A*_2_ symmetry produce nodes along the Γ–M directions, we choose the simplest, *p*-wave term in the toy model, resulting in

8 $$\begin{array}{l}\Delta ({k}_{1},{k}_{2},z)\,=\,{f}_{1}(z){\tau }_{0}[\sin {k}_{1}{{\sigma }}_{y}+\sin {k}_{2}{{\sigma }}_{y,1}-\sin ({k}_{1}+{k}_{2}){{\sigma }}_{y,2}\\ \,+\,\sin ({k}_{1}+2{k}_{2}){{\sigma }}_{x}-\sin (2{k}_{1}+{k}_{2}){{\sigma }}_{x,1}+\sin ({k}_{1}-{k}_{2}){{\sigma }}_{x,2}]({\rm{i}}{{\sigma }}_{y}),\end{array}$$

where

9 $${\sigma }_{x/y,j}={{\mathcal{C}}}_{3}^{j}{\sigma }_{x/y}{{\mathcal{C}}}_{3}^{-j}.$$

Linearizing equation (8) produces the same type of pairing matrix as the *p*-wave term of equation (2). As mentioned above, the amplitude *f*_1_(*z*) = 2 for the two top-most and bottom-most unit cells, whereas *f*_1_ = 0 otherwise. We chose such a large value for the pairing term to enhance the gap along the Fermi arc, thus reducing finite-size effects and enabling us to visualize Majorana states for numerically accessible system sizes.

Finally, to better differentiate between the Fermi arcs on the top and bottom surfaces, we shift the value of *μ* on the bottom-most unit cell (*z* = 0) from *μ* = 2 to *μ* = 1.7. This causes the top and bottom Fermi arcs to occur at different momenta in the slab Brillouin zone, such that they can be more easily visualized.

Extended Data Fig. 2a,b are obtained in a slab geometry consisting of 80 unit cells in the *z-*direction, in which the Cartesian momentum directions are defined as *k*_*y*_ = *k*_1_ and ${k}_{x}=({k}_{1}+2{k}_{2})/\sqrt{3}$. The gap is always computed as the absolute value of the eigenenergy closest to the Fermi level, *E*_F_ = 0.

For each surface Majorana cone, we determine the winding number by using the standard approach of rotating the Hamiltonian to a block off-diagonal form. We use the full slab Hamiltonian, enabling us to determine the winding numbers of Majorana cones on both the top and the bottom surfaces.

To minimize finite-size effects, Extended Data Fig. 2c is obtained for a slab consisting of 1,000 unit cells in the *z-*direction and shows the gap opening in the Majorana cone on the top surface, indicated by an orange arrow in Extended Data Eig. 2b. The Zeeman field is included by adding an onsite term *V*_*z*_*τ*_0_*σ*_*z*_ to the Hamiltonian equation (3).

Extended Data Fig. 4d is reproduced from Fig. 3c and shows the coexistence of bulk Weyl cones, surface Majorana cones and Majorana hinge modes. To reduce the finite-size gap that would otherwise be present in these features, the panel includes results from two separate simulations. The green, red and blue points are obtained in a slab geometry (infinite along both *k*_*x*_ and *k*_*y*_), and consist of 320 unit cells along the *z-*direction. Eigenvalues are computed for different values of *k*_*x*_, in steps of 10^−3^, and plotted as a function of *k*_*y*_ ∈ [1.05, 1.45]. The colour represents the state probability density summed over the bottom half of the slab, meaning for values *z* < 160. The apparently sharp transition between the different colours is a consequence of our plotting choice. Multiple points of different colours are plotted on top of each other, and the only visible colours correspond to those points that are plotted last. The hinge states, shown in black, are then superimposed on the slab band structure plot. The hinge modes are obtained in a prism geometry, infinite along *y*, 40 unit cells along *z* and 300 unit cells along *x*. This is also the geometry of the system that is used in Fig. 3e, where the colour scale denotes the probability density summed over the four states closest to *E* = 0 at *k*_*y*_ = 1.3, as marked by the green arrow in Extended Data Fig. 4d.

### Leading edge, peak and trailing edge positions of the energy distribution curves along the arc of PtBi_2_

**Extended Data Fig. 3:**
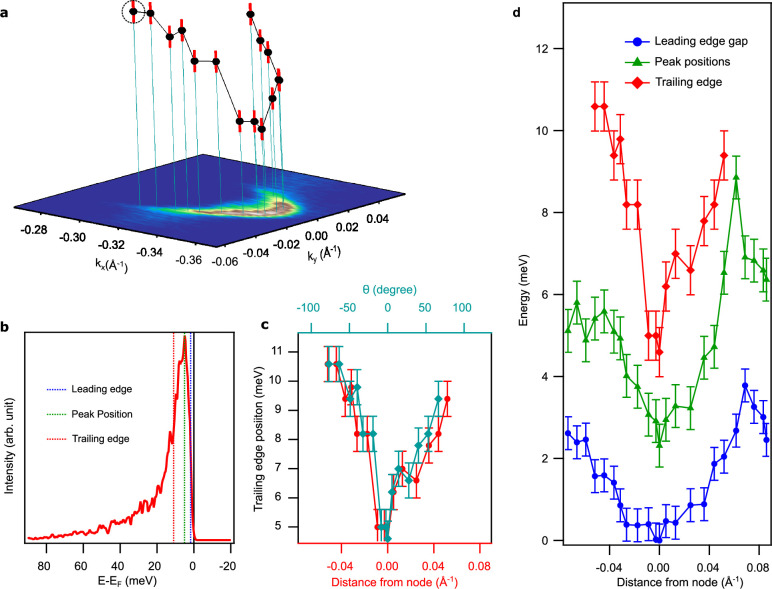
Leading edge, peak positions, and trailing edge data. **(a)** Trailing edge positions across different points of the arc. **(b)** Representative energy distribution curve (EDC) taken at the circled data point in (a) (k_*x*_= –0.30 Å^−1^, k_*y*_= –0.031 Å^−1^), shows the positions of leading edge, EDC peak and trailing edge. **(c)** Trailing edge position as a function of *θ* (deg) and distance from the node along the arc (Å^−1^). **(d)** The leading edge, peak positions, and trailing edge positions as a function of distance from the node (Å^−1^). The energy scale is determined relative to the leading edge position of the nodal EDC.

As mentioned in the main text, compared with the leading edge, the energy distribution curve peak, and more significantly, the trailing edge positions at higher binding energies are more accurate for determining the gap function near the node. In Extended Data Fig. 3a, the trailing edge positions are overlaid on the Fermi surface map for visualization. For comparison, the leading edge, peak and trailing edge positions of the energy distribution curves are plotted as a function of distance from the node in Extended Data Fig. 3d. It is to be noted that the SC gap is plotted with respect to the distance from the node in the calculations (Fig. 3b), whereas the gap function is presented with respect to *θ* in Fig. 2. To draw one-to-one correspondence, we have plotted the trailing edge as a function of both *θ* and distance from the node in Extended Data Fig. 3c. The dip in the trailing edge position near the node is much sharper compared with the leading edge position and agrees more with the gap function obtained from calculations near the node (Fig. 3a).

#### Leading edge, peak and trailing edge positions for BSCCO

**Extended Data Fig. 4:**
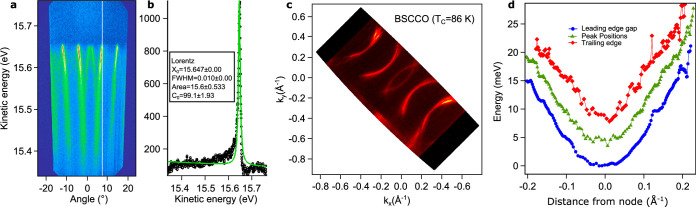
Leading edge, peak position, and trailing edge in BSCCO. **(a)** Energy distribution map (EDM) collected from BSCCO at low temperature. **(b)** The Energy distribution cut (EDC) corresponding to the white like in (a). The Fitting the EDC peak show sharp coherence peak with FWHM of 10 meV. **(c)** Fermi surface map of BSCCO. **(d)** The leading edge, peak position and trailing edge position of the coherence peak of BSCCO as a function of distance from the node.

To demonstrate how the LEG curves are affected by the Fermi function, we have plotted the leading edge, peak and trailing edge positions for BSCCO (bismuth strontium calcium copper oxide), which is a well-established nodal superconductor. Extended Data Fig. 4a–c shows the overall good quality of the collected ARPES data. Similar to PtBi_2_, the trailing edge position of BSCCO harbours a much sharper feature near the node compared with the leading edge and peak positions (Extended Data Fig. 4d).

## Online content

Any methods, additional references, Nature Portfolio reporting summaries, source data, extended data, supplementary information, acknowledgements, peer review information; details of author contributions and competing interests; and statements of data and code availability are available at 10.1038/s41586-025-09712-6.

## Figures and Tables

**Extended Data Table 1:**
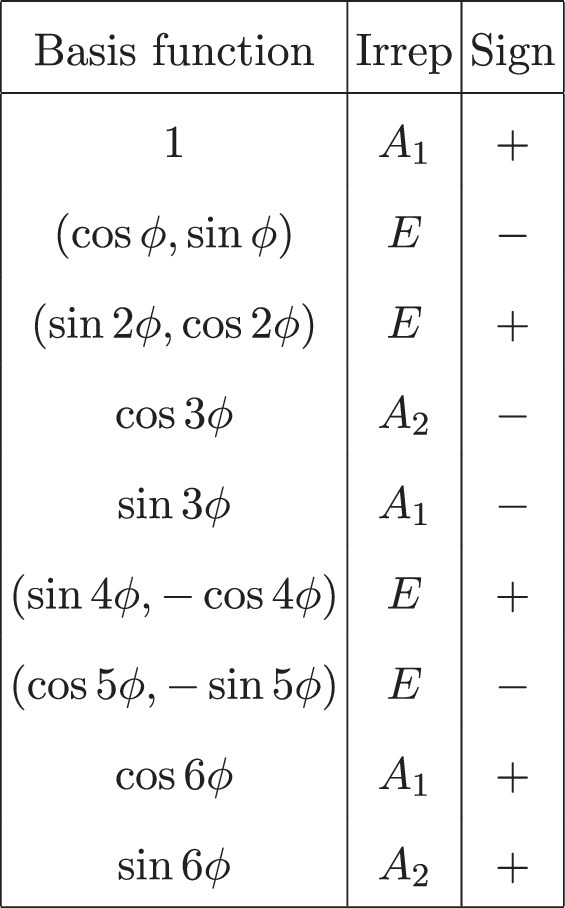
Low-order basis functions of the polar angle *Φ* in momentum space

For the irrep *E*, the first and second component are assigned according to the choice of (*k*_*x*_, *k*_*y*_) as basis functions. For each function or pair of functions, the irrep and the sign under time reversal are given.

**Extended Data Table 2:**
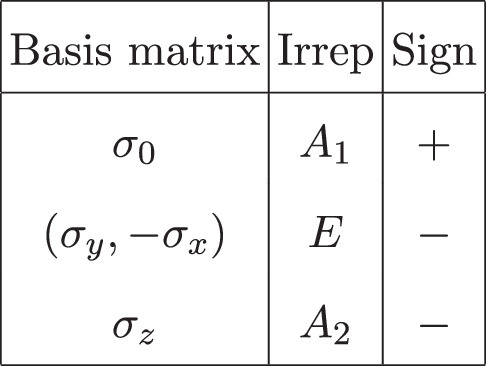
Basis matrices acting on spin space

Each basis matrix is an irreducible tensor operator belonging to the irrep given in the second column. The sign under time reversal is also given.

## Data Availability

The data related to this study are available at Zenodo^43^ (10.5281/zenodo.14283531).

## Code availability

The code used in this study is available at Zenodo^43^ (10.5281/zenodo.14283531).
